# Respiratory Adherence Care Enhancer Questionnaire: Identifying Self-Management Barriers of Inhalation Corticosteroids in Asthma

**DOI:** 10.3389/fphar.2021.767092

**Published:** 2021-12-22

**Authors:** Claire D. Visser, Jip M. Linthorst, Esther Kuipers, Jacob K. Sont, Joyca P. W. Lacroix, Henk-Jan Guchelaar, Martina Teichert

**Affiliations:** ^1^ Department of Clinical Pharmacy and Toxicology, Leiden University Medical Center, Leiden, Netherlands; ^2^ Royal Dutch Pharmacists Association (KNMP), The Hague, Netherlands; ^3^ Community Pharmacy Empel, ‘s-Hertogenbosch, Netherlands; ^4^ Department of Biomedical Data Sciences, Section Medical Decision Making, Leiden University Medical Center, Leiden, Netherlands; ^5^ Department of Digital Engagement, Behavior and Cognition, Philips Research, Eindhoven, Netherlands

**Keywords:** asthma, inhaled corticosteroids (ICS), self-management (self-care), theoretical domains framework (TDF), tailored care, adherence–compliance–persistance

## Abstract

**Introduction:** Suboptimal self-management of inhaled corticosteroids (ICS) in asthma patients is frequently observed in clinical practice and associated with poor asthma control. Driving factors for suboptimal self-management are complex and consist of a range of behavioral barriers (cognitive, affective and practical) with a considerable inter-individual variability. Identification of individual barriers facilitates the use of corresponding behavior change techniques and tailored care to improve asthma treatment outcomes.

**Objective:** This study describes the development and validation of the ‘Respiratory Adherence Care Enhancer’ (RACE) questionnaire to identify individual barriers to self-management of ICS therapy in asthma patients.

**Methods:** The development included: 1) an inventory of self-management barriers based on a literature review, 2) expert assessment on relevance and completeness of this set, linking these barriers to behavioral domains of the Theoretical Domains Framework (TDF) and 3) the formulation of corresponding questions assessing each of the barriers. A cross-sectional study was performed for validation. Primary care asthma patients were invited to fill out the RACE-questionnaire prior to a semi-structured telephonic interview as golden standard. Barriers detected from the questionnaire were compared to those mentioned in the interview.

**Results:** The developed questionnaire is made up of 6 TDF-domains, covering 10 self-management barriers with 23 questions. For the validation 64 patients completed the questionnaire, of whom 61 patients were interviewed. Cronbach’s alpha for the consistency of questions within the barriers ranged from 0.58 to 0.90. Optimal cut-off values for the presence of barriers were determined at a specificity between 67 and 92% with a sensitivity between 41 and 83%. Significant Areas Under the Receiver Operating Curves values were observed for 9 barriers with values between 0.69 and 0.86 (*p*-value <0.05), except for ‘Knowledge of ICS medication’ with an insignificant value of 0.53.

**Conclusion:** The RACE-questionnaire yields adequate psychometric characteristics to identify individual barriers to self-management of ICS therapy in asthma patients, facilitating tailored care.

## Introduction

Asthma is a chronic respiratory disease affecting an estimated 300 million individuals worldwide (GBD 2017 Disease and Injury Incidence and Prevalence Collaborators, 2018). It is a public health problem placing a significant socioeconomic burden on patients, caregivers and healthcare systems (Dharmage et al., 2019; Dierick et al., 2020). Inhaled corticosteroids (ICS) are considered the cornerstone of controller therapy for asthma according to the recommendations of the Global Initiative For Asthma (GINA) guideline (Global Initiative for Ast, 2020). These drugs have the ability to effectively control asthma by suppressing airway inflammation, reducing bronchoconstriction and concomitant asthma symptoms such as breathlessness and wheezing (Barnes, 2010; Global Initiative for Ast, 2020). However, approximately 50% of asthma patients do not follow the prescribed ICS regimen due to factors related to self-management including poor adherence, awareness of the disease or the lacking of practical skills (World Health Organisation, 2021). This phenomenon has been associated with an increased risk of exacerbations, impaired quality of life, hospitalization, and mortality (Bårnes and Ulrik, 2015).

Healthcare professionals interact with asthma patients in the field of respiratory health during consultations or prescription refills and have an important role in providing long-term care for effective self-management. They educate patients on their disease and inhalation medication, provide training on the use of inhalation devices, address patients’ concerns and beliefs in order to guide optimal self-management of ICS therapy (Bridgeman and Wilken, 2021; Murray and O'Neill, 2018). However, identifying and overcoming barriers to self-management of ICS therapy is a major challenge in clinical practice (Murray and O'Neill, 2018). Driving factors are complex and consist of a range of cognitive, affective and practical barriers with a considerable inter-individual variability (Boulet et al., 2012; World Health Organisation, 2021). Healthcare professionals often lack good insight into this multitude of barriers and therefore apply a ‘‘one size fits all’’ approach, frequently focusing on adherence and practical skills without addressing the underlying individual behavioral barriers (Plaza et al., 2016; Dima et al., 2017; Anghel et al., 2019; Toelle et al., 2020). Enabling healthcare professionals with a tool to identify individual barriers to self-management of ICS therapy may be of added value to facilitate tailored care to optimize self-management and treatment outcomes.

Based on psychological behavioral change theories, the Theoretical Domains Framework (TDF) offers a set of domains with associated constructs composed of cognitive, affective, social and environmental factors. This validated framework can be applied to identify individual behavioral challenges and to develop corresponding interventions as a strategy to overcome behavioral barriers (Cane et al., 2012; French et al., 2012). Thus, the TDF is appropriate as a theoretical underpinning for asthma patient behavior, enhancing existing interventions in their effectiveness to overcome individual barriers to self-management of ICS therapy in clinical practice (Allemann et al., 2016; Presseau et al., 2017; Patton et al., 2018).

Hence, the aim of this study was to develop and validate a questionnaire based on the TDF for identification of individual barriers to self-management of ICS therapy in primary care asthma patients: the ‘‘Respiratory Adherence Care Enhancer’’ (RACE) questionnaire.

## Methods

### Study Design

A mixed-methodology approach was conducted in this study to develop and validate the RACE questionnaire (Supplementary 1.1). The development of the questionnaire was based on a literature review. The questionnaire was validated by the assessment of the content and face validity as part of the development phase. To validate the questionnaire on internal consistency and criterion validity of the barriers with asthma patients, a cross-sectional study was performed. Primary care asthma patients were invited to fill out the questionnaire, followed by a telephonic semi-structured interview as a golden standard method within a 2-week period. The study protocol (N19.097) was declared to not fall within the scope of the Dutch Medical Research Involving Human Subjects Act by the Medical Ethics Committee (MEC) of the Leiden University Medical Center (LUMC). The study was approved by the scientific committee of the division of Clinical Pharmacy and Toxicology of the LUMC and was conducted according to the principles of the World Medical Association Declaration of Helsinki. Written informed consent was obtained from all included patients prior to participation.

### Development

#### Systematic Literature Review

A systematic literature review was performed to identify articles published between January 2000 and October 2018 concerning barriers to self-management or adherence of ICS therapy in asthma patients. The search was performed in PubMed and the Web of Science database using a combination of the following keywords and Medical Subject Headings (MeSH): “asthma”, “barriers”, ‘‘self-management’’ and “adherence”. All obtained records were screened by two independent researchers (RM, MT) according to predefined inclusion criteria including ‘‘asthma patients receiving ICS therapy’’ and ‘‘barriers concerning non-adherence, beliefs or self-management’’ to determine their eligibility in a three-stage screening process which consisted of title, abstract- and full-text screening. Duplicates, commentaries, editorials, poster abstracts, letters without original data and publications in other languages other than English or Dutch were excluded. Consensus on discrepancies of the included articles was achieved through discussion. Subsequently, barriers to self-management or adherence of ICS therapy were extracted and summarized according to the TDF.

#### Content and Face Validity

An expert panel was consulted in two Delphi rounds to assess the clinical relevance, completeness and feasibility of the set of extracted barriers and to cluster these barriers into TDF-domains. Pharmacists of the Special Interest Group (SIG) Lung from the Royal Dutch Pharmacists Association (KNMP) and General Practitioners (GP) were invited to this panel. In round 1, participants were requested to rate the barriers on the abovementioned with a scale from 0 to 10 (low to high). In round 2 participants reappraised the results from round 1 through the same method. Subsequently, questions were formulated to address the barriers identified (JML, MT). Questions were framed as negative and positive statements to limit social-desirability bias (Larson, 2018). For the responses, a 5-point Likert scale was provided ranging from complete disagreement to complete agreement. Questions on medication use and the validated ‘‘Control of Allergic Rhinitis and Asthma Test’’ (CARAT10) were added to assess asthma control (Fonseca et al., 2010). The final questionnaire was tested for face validity by pharmacists of the SIG Lung and researchers with experience in the development of questionnaires, to assess the readability, feasibility and comprehensive understanding of the questions.

Forward-backward translation was performed on the original Dutch version of the RACE questionnaire, providing an English version. Forward translation was conducted by a native English speaker and backward translation by a native Dutch speaker. These two processes were performed independently of each other, after which the two Dutch versions were compared and discrepancies were resolved in a consultation session.

### Validation

For the assessment of the internal consistency and criterion validity of the barriers, a cross-sectional study was performed in primary care asthma patients. As there were no other comparable measuring tools available, all respondents to the RACE questionnaire were invited for semi-structured interviews. The barriers identified from these interviews were considered as golden standard to validate the barriers as detected from the scores on the RACE questionnaire.

#### Study Participants

A total of 20 community pharmacists from the SIG lung and the internship database of the Leiden University were invited to each recruit 10 asthma patients with ICS dispensing’s between October and December 2019. The aim of this study was to include 100 patients at a non-response rate of 50%. Patients were eligible for inclusion when ≥18 years, diagnosed with asthma and treated with ICS according to dispensing data present in the pharmacy information systems. Patients with Chronic Obstructive Pulmonary disease (COPD), suspicions hereof or diagnosed with other significant lung diseases were excluded. Likewise, the incapability to speak, write and comprehend the Dutch language were exclusion criteria.

#### Data-Collection

An online version of the RACE questionnaire was built within the facilities of Castor Electronic Data Capture (EDC) to safeguard the collected data in the LUMC surroundings according to the Data Protection Act. If requested by the patient, a paper version of the questionnaire was provided.

Semi-structured interviews were performed with the responders to the questionnaire. An interview guide was developed with comparable components, prompts and recommendations as presented in the TDF-based interview topic guides of earlier studies (Supplementary 2.1) (Presseau et al., 2017; DeJonckheere and Vaughn, 2019). This method was applied to elicit and encourage patients to provide overt information concerning their feelings, thoughts and beliefs on the TDF domains and barriers. To retain the objectivity of the provided information in the interviews, the components of the questionnaire were presented in a different order and posed through an open dialogue approach. The interviews were conducted in the Dutch language by a member of the research team (JML) who did not have any prior contact with the participants and no access to the scores of the questionnaire. The interviews were recorded, transcribed verbatim and independently categorized for barriers with the aid of a predefined coding framework by two other members of the research team (CV, MT). This framework was developed to provide a clear distinction between the presence or absence of each barrier, ensuring a complete and comprehensive understanding of existing barriers in the participants (Supplementary 2.2). The interrater reliability was assessed between the coding appraisals. Any discrepancies of the coded data were resolved through discussions. The recordings and transcripts were safeguarded within the LUMC password-secured surroundings.

Patient’s judgements on the questionnaire were queried as last question in the semi-structured interview and taken into consideration in the final RACE questionnaire.

#### Data-Analysis

Descriptive analysis was performed to describe demographic characteristics and the distribution of barriers of the overall included sample population.

Cronbach’s alpha test was used to determine the internal consistency of the questions within each barrier. Values between 0.6 and 0.7 were considered as acceptable and values between 0.7 and 0.9 indicated a good level of reliability (Taber, 2018). Cohen’s kappa statistic was performed to assess the inter-rater reliability between the coding appraisals of the semi-structured interview data. Values of at least 0.6 were considered acceptable (Landis and Koch, 1977).

The identification of cut-off values for the presence of barriers were determined for optimal specificity and sensitivity values by receiver operating characteristic (ROC) analysis. The sum scores of the questions per barrier obtained from the RACE questionnaire and the binary outcomes from the interviews were used for the ROC curves. Furthermore, criterion validity was assessed with the optimal sensitivity and specificity values. If necessary, higher specificity was preferred to higher sensitivity to reduce the number of false positives, maintaining practicability in clinical practice. Additionally, the area under the curve (AUROC) was calculated per barrier to determine the ability of the barrier scores to discriminate between barrier presence or absence by comparing the AUROC values to a chance value of 0.5. Two-tailed *p*-values ≤ 0.05 were considered significant.

Statistical analyses were performed using SPSS statistical software (version 26.0, IBM corp., Armonk, NY).

## Results

### Development

**FIGURE 1:**
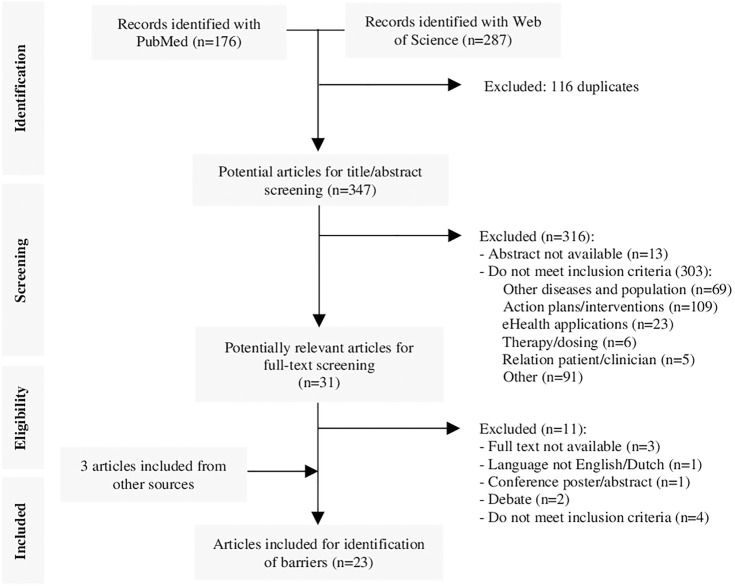
Systematic literature review flowchart.

A total of 347 potentially relevant articles were identified from the systematic literature review, of which 316 were excluded based on their titles and/or abstract. Full-text screening was conducted on the remaining 31 articles, resulting in the exclusion of 11 articles and the addition of 3 articles identified from reference lists. The final review included 23 articles (Figure 1) containing 32 barriers to self-management of ICS therapy in asthma patients, which could be related to 8 TDF-domains. The clinical relevance of the extracted barriers and their relation to the TDF-domains were assessed by a panel of 9 pharmacists from the SIG Lung and 1 GP in Delphi-rounds, resulting in the addition of 1 barrier and exclusion of 13 barriers and 2 TDF-domains. The final version of the RACE questionnaire included 6 TDF domains, covering 10 self-management barriers with 23 corresponding questions (Table 1). The RACE questionnaire is provided as additional material (Table 4).

**TABLE 1:** RACE questionnaire construct for the identification of self-management barriers.

TDF-domains	Barriers	Questions (n)^a^	Total score^b^
Knowledge	Knowledge of asthma	2	0–8
	Knowledge of ICS medication	3	0–12
Beliefs about consequences	Expectations of ICS medication	2	0–8
	Experience of side-effects	2	0–8
Emotion	Concerns about ICS inhaler	3	0–12
	Social discomfort of inhaling with ICS in public	1	0–4
Skills	Understanding and application of ICS inhaler techniques	3	0–12
Memory, attention and decision process	(Un)Conscious adherence to prescribed ICS medication regimen	3	0–12
	Shared treatment decision making	3	0–12
Behavioural regulation	Existence of structure in ICS medication intake	1	0–4

a Responses to the questions are provided on a 5-point Likert scale with the following options: I disagree completely, I disagree mostly, I agree somewhat, I agree mostly and I agree completely. Scores to these options vary from 0 to 4 or 4 to 0 dependent on the question.

b Sum scores were computed per barrier by adding the scores of the corresponding questions. Total scores achievable for a barrier differ for the number of questions included, with a maximum of 4 points to be obtained per question.

Abbreviations: RACE: respiratory adherence care enhancer; TDF: theoretical domains framework; ICS: inhaled corticosteroids.

### Validation

#### Participating Patients

Eligible asthma patients were invited by 16 community pharmacies. An informed consent was provided by 73 patients of whom 8 patients did not respond to the RACE questionnaire and 1 patient was excluded due to mentioning diagnosis with COPD during the interview. Therefore, 64 patients were enrolled in this study. Semi-structured interviews were conducted in 63 of these patients of which 2 interviews were excluded due to an unclear record and 1 interview was excluded due to record failure. This resulted in 61 patients available for assessment of criterion validity.

**FIGURE 2:**
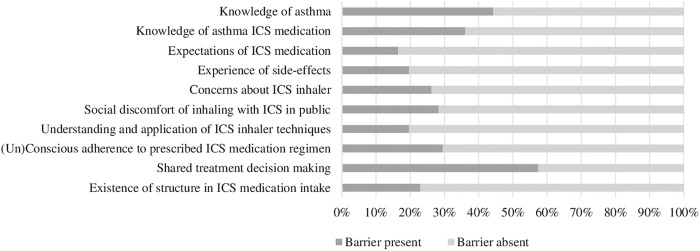
Percentages of barriers present identified from the semi-structured interviews (*n* = 61). Abbreviations: ICS: Inhaled Corticosteroids.

Demographic characteristics of the included patients are presented in Table 2. Of the 64 patients 70.3% were female. With regard to their ICS therapy, 39.1% of the patients were reported to use pressurized metered dose inhalers (pMDI’s) and 59.4% dry powder inhalers (DPI’s). The majority of the patients used their ICS on a daily basis as maintenance therapy (90.6%) and a small number of patients as reliever therapy (9.4%). Adequate asthma control, as established by a CARAT-score of >24, was present in 39.1% of the patients. The presence of the barriers ranged between 16.4 and 57.4% according to the semi-structured interviews (Figure 2). The majority of the barriers detected concerned ‘Shared treatment decision making’ (57.4%), ‘Knowledge of asthma’ (44.3%) and ‘Knowledge of ICS medication’ (36.1%).

**TABLE 2:** Demographic characteristics of the included study participants (n = 64).

**Variables**	**Number (Percentages)**
Gender (Female)	45 (70.3%)
Type of inhaler used	
pMDI	25 (39.1%)
DPI	38 (59.4%)
Unknown	1 (1.6%)
Reliever ICS therapy	6 (9.4%)
Maintenance ICS therapy	58 (90.6%)
Adequate asthma control	25 (39.1%)

Abbreviations: pMDI: pressurized Metered Dose Inhaler; DPI: dry powder inhaler; ICS: inhaled corticosteroids.

#### Psychometric Characteristics

Cronbach’s alpha values ranged from 0.58 to 0.89 (Table 3). Three barriers were considered good Cronbach’s alpha values, including ‘Expectations of ICS medication’ (0.89), ‘Concerns about ICS inhaler’ (0.80) and ‘(Un)conscious adherence to prescribed ICS medication regimen’ (0.77). The barriers ‘Knowledge of asthma’ (0.67), ‘Knowledge of ICS medication’ (0.65), ‘Experience of side-effects’ (0.66) and ‘Shared treatment decision making’ (0.66) were considered acceptable. For the barrier ‘Understanding and application of ICS inhaler techniques’ Cronbach’s alpha was just below the acceptable range (0.58). Cohen’s kappa values ranged between 0.35 and 0.96 for the inter-rater reliability of the coding of the barriers from the interviews, with a total of 5 barriers above 0.6 (Table 5).

**TABLE 3:** Psychometric characteristics of the self-management barriers on the RACE questionnaire.

TDF-domain	Barrier	Reliability	Criterion validity				
		Cronbach’s α^a^	Cut-off	Sensitivity (%)	Specificity (%)	AUROC	*p*-value^b^
Knowledge	Knowledge of asthma	0.67	3.5	59.3	70.6	0.70	**0.007**
	Knowledge of ICS medication	0.65	3.5	40.9	71.8	0.53	0.724
Beliefs about consequences	Expectations of ICS medication	0.89	3.5	60.0	82.4	0.81	**0.002**
	Experience of side-effects	0.66	2.5	58.3	91.8	0.73	**0.014**
Emotion	Concerns about inhaler medication	0.80	3.5	75.0	73.3	0.77	**0.002**
	Social discomfort of inhaling with ICS in public	N.A^c^	1.5	47.1	83.7	0.69	**0.022**
Skills	Understanding and application of ICS inhaler techniques	0.58	2.5	50.0	67.3	0.69	**0.042**
Memory, attention and decision process	(Un)conscious adherence to prescribed ICS medication regimen	0.77	3.5	83.3	79.1	0.86	**0.000**
	Shared treatment decision making	0.66	5.5	65.7	73.1	0.76	**0.001**
Behavioural regulation	Existence of structure in ICS medication intake	N.A^c^	1.5	64.3	89.4	0.80	**0.001**

a Cronbach’s alpha values between 0.6 and 0.7 indicate an acceptable level of reliability and values between 0.7 and 0.9 indicate a good level of reliability.

b *p*-value ≤ 0.05 was set as statistically significant for the assessment of the accuracy of the barriers to discriminate between the presence and absence of the barrier and are printed in bold.

c The internal consistency test was not applicable (N.A.) as only one question was included in the barrier.

Abbreviations: RACE: respiratory adherence care enhancer; TDF: theoretical domains framework; ICS: inhaled corticosteroids; AUROC: area under the receiver operating characteristic; N.A.: not applicable.

**TABLE 4:** Description of the RACE questionnaire identifying individual barriers to self-management of ICS therapy.

TDF-domains	Barriers	Questions	Score^a^	Total score^ab^	Interpretation
Knowledge	Knowledge of asthma	1a. I know what triggers an asthma attack	4–0	0–8	BA:0–3
		1b. I know how to prevent an asthma attack	4–0		BP: 4–8
	Knowledge of ICS medication	2a. I know that my anti-inflammatory inhaler reduces the swelling of the lining in my airways	4–0	0–12	BA: 0–3
		2b. I know that my anti-inflammatory inhaler improves the condition of my airways	4–0		BP: 4–12
		2c. I know that my anti-inflammatory does not provide quick relief of my asthma symptoms but it tackles the cause of my asthma^c^	4–0		
Beliefs about consequences	Expectations of ICS medication	3a. I need my anti-inflammatory inhaler to keep my asthma stable	4–0	0–8	BA: 0–3
		3b. I need my anti-inflammatory inhaler to prevent my asthma from getting worse	4–0		BP: 4–8
	Experience of side-effects	4a. I experience side-effects from my anti-inflammatory inhaler	0–4	0–8	BA: 0–2
		4b. The side-effects of my anti-inflammatory inhaler reduce my daily functioning	0–4		BP: 3–8
Emotion	Concerns about ICS inhaler	5a. I am concerned about possible side-effects from my anti-inflammatory inhaler	0–4	0–12	BA: 0–3
		5b. I am concerned about long-term side-effects from my anti-inflammatory inhaler	0–4		BP: 4–12
		5c. I dread having to inhale regularly with an anti-inflammatory inhaler for my asthma	0–4		
	Social discomfort of inhaling with ICS in public	6. I prefer not to use my inhaler in public^d^	0–4	0–4	BA: 0–1
					BP: 2–4
Skills	Understanding and application of ICS inhaler techniques	7a. I understand the instructions on how to use my anti-inflammatory inhaler	4–0	0–12	BA: 0–2
		7b. With the instructions I am capable of using my anti-inflammatory inhaler properly	4–0		BP: 3–12
		7c. I find it difficult to inhale properly with my anti-inflammatory inhaler	0–4		
Memory, attention and decision process	(Un)conscious adherence to prescribed ICS medication regimen	8a. I use my anti-inflammatory inhaler every day	4–0	0–12	BA: 0–3
		8b. I use my anti-inflammatory inhaler as prescribed by my healthcare provider	4–0		BP: 4–12
		8c. Sometimes I forget to use my anti-inflammatory inhaler	0–4		
	Shared treatment decision making	9a. My healthcare provider (doctor, nurse, pharmacist or lung specialist) has discussed with me in which way my asthma can best be treated	4–0	0–12	BA: 0–5
		9b. My healthcare provider (doctor, nurse, pharmacist or lung specialist) has asked me which type of inhaler I prefer	4–0		BP: 6–12
		9c. My healthcare provider (doctor, nurse, pharmacist or lung specialist) has discussed with me how I can best use my anti-inflammatory inhaler to prevent an asthma attack	4–0		
Behavioral regulation	Existence of structure in ICS medication intake	10. I inhale at a fixed time of the day	4–0	0–4	BA: 0–1
					BP: 2–4

a Responses to the questions are provided on a 5-point Likert scale with the following options: I disagree completely, I disagree mostly, I agree somewhat, I agree mostly and I agree completely. Scores to these options vary from 0 to 4 or 4 to 0 dependent on the question.

b Sum scores per barrier can be computed by adding the scores of the responses on the questions per barrier.

c Additional adjustments have been implemented on this question after validation. Former question: I know how to use my ICS inhaler.

d Additional adjustments have been implemented on this question after validation. Former question: I am embarrassed to use my inhaler in public.

Abbreviations: RACE: respiratory adherence care enhancer; TDF: theoretical domains framework; ICS: inhaled corticosteroids; BA: barrier absent; BP: barrier present.

**TABLE 5:** The inter-rater reliability test results of the appraisals on the presence/absence of self-management barriers according to the interview coding’s of two analysts.

TDF-domains	Barriers	Cohen’s kappa^a^
Knowledge	Knowledge of asthma	**0.77**
	Knowledge of ICS medication	0.35
Beliefs about consequences	Expectations of ICS medication	0.52
	Experience of side-effects	0.54
Emotion	Concerns about ICS inhaler	0.41
	Social discomfort of inhaling with ICS in public	**0.88**
Skills	Understanding and application of ICS inhaler techniques	**0.58**
Memory, attention and decision process	(Un)Conscious adherence to prescribed ICS medication regimen	**0.68**
	Shared treatment decision making	**0.64**
Behavioral regulation	Existence of structure in ICS medication intake	**0.96**

a Cohen’s kappa values higher or equal to 0.6 were considered acceptable and are printed in bold.

Abbreviations: RACE: respiratory adherence care enhancer; TDF: theoretical domains framework; ICS: inhaled corticosteroids; AUROC: area under the receiver operating characteristic.

**FIGURE 3:**
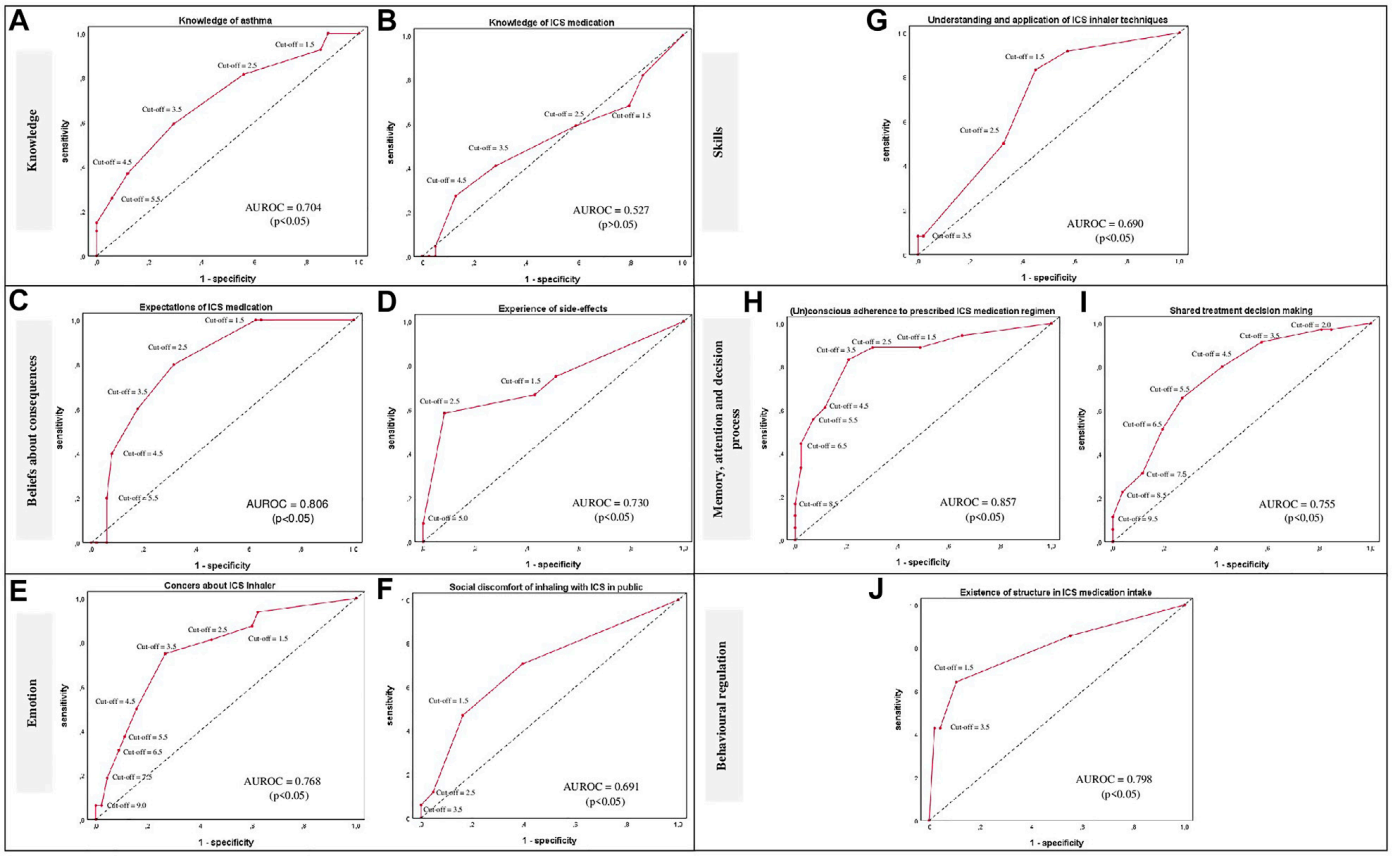
ROC curves per barrier **(A–J)** are provided per TDF-domain for the classification of patients in presence or absence of a self-management barrier using various cut-off points of the sum scores per barrier. The ROC curves are presented as red lines. The diagonal dashed line represents the line that is no better than chance at discriminating between the presence or absence of a self-management barrier. Adjuvant AUROC values are presented per graph providing an indication of the accuracy of the scores at discriminating between the presence or absence of a self-management barrier. Corresponding *p*-values ≤ 0.05 were considered statistically significant; Abbreviations: ROC: Receiver operating characteristic; TDF: Theoretical Domains Framework; AUROC: Area under the ROC; ICS: Inhaled Corticosteroids.

Optimal cut-off values for the presence of a barrier were determined at specificity values between 67.3 and 91.8% with sensitivity values between 40.9 and 83.3% for the 10 barriers (Table 3). In addition, significant AUROC values were determined for the barriers ranging between 0.69 and 0.86 (*p*-value <0.05), except for the barrier ‘Knowledge of ICS medication’ with an AUROC value of 0.53 (Figure 3). Further investigation of this barrier revealed a poorly comprehensible question on knowledge of the difference between bronchodilator and anti-inflammatory inhalers which has been revised. Also, after evaluation of the patient’s judgements, the terminology for ‘shame’ was adjusted in the question corresponding to the barrier ‘Social discomfort of inhaling with ICS in public’ (Table 4).

## Discussion

This study described the development and validation of the RACE questionnaire to identify individual barriers to self-management of ICS therapy in primary care asthma patients.

The concise set of barriers addressed has shown associations with decreased self-management, adherence and poor treatment outcomes with ICS therapy in asthma patients (Ponieman et al., 2009; Wilson et al., 2010; Dima et al., 2015; Foster et al., 2017; George and Bender, 2019). Clinical relevance of these barriers has also been verified by a panel of experts during the development of the questionnaire. At present multiple questionnaires are available for asthma patients, mainly emphasizing on barriers related to (un)conscious adherence and side-effects (Plaza et al., 2016; Dima et al., 2017; Toelle et al., 2020). However, these questionnaires often consider adherence as a single outcome without acknowledging that adherence is influenced by a set of underlying behavioral barriers that need to be overcome to optimize adherence. Other questionnaires as the Self-Management Screening (SeMaS) questionnaire signal essential individual barriers to self-management (Eikelenboom et al., 2015). Nevertheless, the contents of this questionnaire are generic and aimed at a range of chronic conditions. The Nijmegen Clinical Screening Instrument (NCSI) for patients with COPD also provides a tailored approach measuring disease-specific characteristics that determine their health status (Vercoulen, 2012). Yet, these questionnaires do not highlight specific underlying barriers concerning inhalation therapy or asthma requiring behavior change. Moreover, interventions targeting self-management or adherence of ICS therapy lack a theoretical underpinning which may clarify their disappointing effectiveness (Normansell et al., 2017). The applied TDF in the RACE questionnaire may therefore aid in the identification of individual behavioral challenges and support matching interventions with behavior change techniques (BCTs) (Cane et al., 2012; French et al., 2012). This may enable healthcare professionals to provide tailored care in a multidisciplinary setting with barrier-specific interventions to overcome or prevent suboptimal self-management of ICS therapy. The study of Patton et al. (2018) has demonstrated potential for the use of TDF to identify and overcome non-adherence to multiple medications in elderly patients.

The RACE questionnaire has shown acceptable to good reliability for the internal consistency of the questions per barrier, except for the barrier ‘Understanding and application of ICS inhaler techniques’ with a value just below the acceptable range. These observed values are in conformity with previous studies addressing comparable screening questionnaires with multiple constructs (Cramm et al., 2012; Eikelenboom et al., 2015). Additionally, any variation in internal consistency of the barriers can be explained by differences in the extent of heterogeneity of the corresponding questions per barrier and the limited number of questions presented per barrier (Tavakol and Dennick, 2011). Whereas the RACE questionnaire was developed to receive relevant information at a concise number of questions, minimizing respondent burden (Turner et al., 2007).

The RACE questionnaire is developed as tool to complement the usual care for asthma patients. It is intended for consultations between patients and healthcare professionals to identify potential barriers to self-management of ICS therapy, facilitating tailored care to improve self-management and treatment outcomes. In this context, higher specificity values were aimed at for the barriers to avoid high false positives, partly at the expense of sensitivity. The barriers ‘Knowledge of asthma medication’, ‘Social discomfort of ICS inhaler in public’ and ‘Understanding and application of ICS inhaler techniques’ presented the lowest sensitivity values with values between 40.9 and 50.0%. However, this does not affect their usefulness and still provides the opportunity to identify approximately half of the asthma patients who have these barriers, that might otherwise remain unnoticed. The accuracy of the questionnaire to discriminate between the presence or absence of the barriers was significant, except for ‘Knowledge of ICS medication’. This may be due to an identified incomprehensible question which has been adjusted in the final RACE questionnaire, requiring further validation.

The personal barrier profiles obtained from the RACE questionnaire should be discussed in consultations between patients and their healthcare professionals. Moreover, this tool might facilitate multidisciplinary cooperation on the provision of tailored interventions, joining the efforts of healthcare professionals. Completion of the questionnaire by the patient might take place prior to the consultation and can be used to monitor potential self-management barriers, behavior change and disease control. Further research will focus on an additional guide with interventions to overcome identified barriers.

There are also some potential limitations in this study which need to be addressed. First, patients were not part of the development of the RACE questionnaire. However, the experts involved were all active in clinical practice. Second, the validation of the questionnaire was performed in a voluntary sample of asthma patients which may not be representative for all asthma patients. Nevertheless, a variability in asthma control, ICS inhaler types and self-management barriers were observed. Also, the possibility of the inclusion of a convenience sample of asthma patients is kept to a minimum as community pharmacists involved are members of special interest and/or university groups and are eager to improve care for lung patients, therefore being more inclined to an objective approach and acknowledging potential pitfalls. Third, less patients than intended could be included in the validation. Yet, these numbers were sufficient to obtain statistically significant AUROC values for nearly all barriers. Fourth, the semi-structured interviews as golden standard for the criterion validity may contain a level of subjectivity. However, other comparable measurements were not available, which was the reason for conducting this study. To retain the objectivity of these interviews, an interview guide was used to encourage patients to provide overt information about their feelings, thoughts and beliefs (Presseau et al., 2017; DeJonckheere and Vaughn, 2019). In this guide, questions were presented in a different order in comparison to the RACE questionnaire. To assure the reliability of the data collected and analyzed, qualitative research standards were applied (Edwards and Holland, 2013).

## Conclusion

The newly developed RACE questionnaire yields adequate psychometric properties for the identification of individual barriers to self-management of ICS therapy in primary care asthma patients. It is therefore ready to be applied in consultations, providing insights into the multitude of barriers that can prevent optimal ICS use in patients. As these barriers are based upon a theoretical underpinning, the next step is to address and overcome these barriers in consultations with tailored advise from healthcare professionals. Subsequently, these efforts should become visible in improved self-management and disease stability of asthma patients.

## Data Availability

The raw data supporting the conclusions of this article will be made available by the authors, without undue reservation.
