# Supplementary material for: Respiratory Adherence Care Enhancer Questionnaire: Identifying Self-Management Barriers of Inhalation Corticosteroids in Asthma
Source: Front Pharmacol. 2021 Dec 22;12:767092. doi: 10.3389/fphar.2021.767092 (PMC8729223; doi:10.3389/fphar.2021.767092)
Supplement: Supplementary file 1 [file Table1.docx]

# Supplementary tables

## Semi-structured interview guide

| **Introduction** | | |
| --- | --- | --- |
| - Provide a brief explanation on the research - Provide a brief explanation on the purpose of this interview - Obtain an oral informed consent for recording of the interview | | |
| **Start semi-structured interview** | | |
| **TDF-domain: Knowledge** | | |
| **Barrier: Knowledge of asthma** | | |
| You are receiving treatment for asthma. What can you tell me about asthma? | | |
| *Prompts* | | - How does asthma occur? - What causes asthma (e.g. name a few triggers)? - What is the progression of asthma (e.g. reversibility of symptoms, quality of life)? - How do you prevent worsening of asthma (e.g. stop smoking, avoid triggers)? |
| **TDF-domain: Memory, attention and decision process** | | |
| **Barrier: Shared treatment decision making** | | |
| How did the decision come about to treat your asthma with an anti-inflammatory inhaler? | | |
| *Prompts* | | - What is your opinion on the information your doctor has provided about the treatment to tackle this condition, the type of inhaler and the dosage? - Were you involved in the decision process of the chosen treatment? - Were your complaints and wishes acknowledged and taken into consideration? |
| **TDF-domain: Knowledge** | | |
| **Barrier: Knowledge of ICS medication** | | |
| What can you tell me about the effects of this medication for your asthma? | | |
| *Prompts* | - What type of information did you receive about your anti-inflammatory inhaler? - What is the function of this drug for asthma (e.g. the effects are not directly noticeable unlike a bronchodilator)? | |
| **TDF-domain: Beliefs about consequences** | | |
| **Barrier: Expectations of ICS medication** | | |
| What expectations do you have from your anti-inflammatory inhaler on your asthma?  What effects does your anti-inflammatory drug have on your asthma?  What will happen if you stop taking your anti-inflammatory inhaler? | | |
| *Prompts* | - How important is your anti-inflammatory inhaler for you? - Are you positive or negative towards using your anti-inflammatory inhaler? | |
| **TDF-domain: Skills** | | |
| **Barrier: Understanding and application of ICS inhaler techniques** | | |
| Having trouble with the application of the inhaler is often recognized. Are the inhaler techniques and application hereof discussed with you by your doctor or pharmacist? | | |
| *Prompts* | - What type of inhaler device do you use? 🡪 Consider questions on important factors corresponding to type of inhaler device:  Dry powder inhaler (DPI): emphasis lies on strong inhalation  Pressurized Metered-dose inhaler (pMDI): emphasis lies on the hand-lung  coordination  Emphasis lies on length of holding breath: at least 10 seconds  Emphasis lies on tilting the head back slightly. - Do you endure any difficulties during inhalation? - What makes inhalation difficult to execute for you? | |
| **TDF-domain: Beliefs about consequences** | | |
| **Barrier: Experience of side-effects** | | |
| All drugs used for any type of condition can cause side-effects, varying per patient.  Are you experiencing any side-effects at the moment?  Are there side-effects that you are concerned about? What type of side-effects are you concerned about? | | |
| *Prompts* | - How do these side-effects affect your daily life? - What concerns do you have? | |
| **TDF-domain: Behavioral regulation** | | |
| **Barrier: Existence of structure in ICS medication intake** | | |
| Agreements may have been made about the use of your anti-inflammatory inhaler with your doctor. How do you need to use your anti-inflammatory inhaler according to your doctor or pharmacist?  Many people find it difficult to take their inhaler daily. How does this work out for you?  Do you have a set time to inhale your anti-inflammatory drug? How do you ensure that you use your anti-inflammatory at a fixed time? | | |
| *Prompts* | - Can you recall moments when it is difficult to comply to this dosing regimen? - Have you made it a habit to inhale regularly with your anti-inflammatory inhaler? - Are there any medication reminders that help you comply to the dosing regimen at fixed moments in time? What are these? - What stimulants/factors encourage you to comply? | |
| **TDF-domain: Emotion** | | |
| **Barrier: Social discomfort of inhaling with ICS in public** | | |
| It may be inconvenient for some people to use their inhaler in public. What is your experience on this? | | |
| *Prompts* | - How do you proceed when becoming breathlessness in public? - How does your environment respond when you start inhaling in public? - Does the usage of your inhaler provide you comfort or stress/anxiety in public? - What concerns do you have? - Do you find it unpleasant to inhale (with a space chamber) in public? Why is this? | |
| **TDF-domain: Memory, attention and decision process** | | |
| **Barrier: (Un)conscious adherence to prescribed ICS medication regimen** | | |
| It can occur that at any time you consciously or unconsciously do not use your inhaler. Can you remember a moment when you consciously or unconsciously did not administer your inhaler? | | |
| *Prompts* | - What is/was the reason for not administering your inhaler ? - In case the participant never forgets his/her inhaler: Do you have any tips for other people on how to achieve this? | |
| **Closing of interview** | | |
| - Obtain opinion on filling out the RACE questionnaire (e.g. level of difficulty, comprehensibility, timeframe) - Provide brief information on follow-up procedures of the research and interview | | |
