# Supplementary material for: Respiratory Adherence Care Enhancer Questionnaire: Identifying Self-Management Barriers of Inhalation Corticosteroids in Asthma
Source: Front Pharmacol. 2021 Dec 22;12:767092. doi: 10.3389/fphar.2021.767092 (PMC8729223; doi:10.3389/fphar.2021.767092)
Supplement: Supplementary file 2 [file Table2.docx]

## Interview coding framework

| **TDF-domain: Knowledge** | | |
| --- | --- | --- |
| **Barrier: Knowledge of asthma** | | |
| *Coding components* | | *Interpretation guide* |
| - Knowledge about the causes/triggers of asthma attacks (patient specific):   - Patient can name specific triggers:     - Allergic triggers (++); non-allergic triggers (++) or medicine use e.g. types of painkillers; NSAID’s (+). | | Mentions one or more specific (non-)allergic triggers: ++  Mentions medicine: +  Unclear/some resemblance: +  Does not mention or know: -  Min. score: -; Max. score: ++. |
| - Knowledge about how to prevent asthma attacks (patient specific):   - Patient can specifically name one or more of the following (++):     - No smoking; daily exercise; tackling obesity; avoiding triggers; treatment of other allergies causing nasal symptoms; administering asthma medication to keep asthma under control. | | Mentions one or more: ++  Unclear/some resemblance: + Does not mention or know: -  Min. score: -; Max. score: ++ |
| - Knowledge about the pathophysiology of asthma in the airways:   - Patient can specifically name one or more of the following:     - Hypersensitive to triggers (++); lining of the airways become inflamed/swells/produces more mucus (++); airways are narrowed from time to time (+); less air/difficulty breathing/breathlessness (+); contraction of muscles around airways (+). | | Mentions one or more: + or ++ dependent on answer given.  Unclear/some resemblance: +  Does not mention or know: -  Min. score: -; Max. score: ++ |
| *Final interpretation* | | *Total score* |
|  | Barrier not present: 5-6 plusses. | 0-6 plusses |
|  | Barrier present: 0-4 plusses. |  |
| **Barrier: Knowledge of ICS medication** | | |
| *Coding components* | | *Interpretation guide* |
| - Knowledge about the mechanism of ICS:   - Patient can specifically name one or more of the following (++):     - Anti-inflammatory/anti-swelling of the airways or mucous membranes/reduce mucus production; protect the airways against triggers that cause breathlessness/react less violently to triggers. | | Mentioning one or more: ++  Unclear/comparable answer: +  Not mentioned/does not know: -  Min. score: -; Max. score: ++ |
| - Knowledge about the effects of ICS on asthma:   - Patient can specifically name one or more of the following (++):     - Improvement of the condition of the lungs; number of asthma attacks are reduced; reducing asthma complaints or symptoms/receiving more air. | | Mentioning one or more: ++  Unclear/comparable answer: +  Not mentioned/does not know: -  Min. score: -; Max. score: ++ |
| - Knowledge about the difference between anti-inflammatory and reliever medication:   - Patient can specifically name one or more of the following (++):     - Reliever medication: Short acing bronchodilator/quick relief; works within minutes; designed to stop an acute attack of breathlessness; emergency medication/temporary solution for asthma complaints/symptoms; used for performing activities e.g. sports.     - Anti-inflammatory medication:   Maintenance/preventive; does not work immediately/takes a few weeks; regular use for preventive/stable/long-term effect; reduced administration of reliever medication due to anti-inflammatory medication. | | Mentioning one or more: ++  Unclear/comparable answer: +  Not mentioned/does not know: -  Min score: -; Max. score: ++ |
| *Final interpretation* | | *Total score* |
|  | Barrier not present: 5-6 plusses. | 0-6 plusses |
|  | Barrier present: 0-4 plusses. |  |
| **TDF-domain: Beliefs about consequences** | | |
| **Barrier: Expectations of ICS medication** | | |
| *Coding components* | | *Interpretation guide* |
| - Expectations about maintaining stable asthma with ICS:   - Patient can specifically name one or more of the following (++):     - Reduction of long-term asthma symptoms: Providing more room to breathe/breathe easily/get more air; preventing breathlessness; improve condition of the lungs; reduce number of asthma attacks. | | Mentioning one or more: ++  Unclear/comparable answer: +  Not mentioned/does not know: -  Min. score: -; Max. score: ++ |
| - Expectations about worsening of asthma without ICS:   - Patient can specifically name one or more of the following (++):     - The airways are no longer protected against triggers that cause breathlessness; asthma symptoms return; long-term consequences (airway remodelling: chronical irreversible change in structure of the airways which cannot be reversed with ICS; consequences: chronic breathlessness or cough). | | Mentioning one or more: ++  Unclear/comparable answer: + Not mentioned/does not know: -  Min. score: -; Max. score: ++ |
| *Final interpretation* | | *Total score* |
|  | Barrier not present: 3-4 plusses. | 0-4 plusses |
|  | Barrier present: 0-2 plusses. |  |
| **Barrier: Experience of side-effects** | | |
| *Coding components* | | *Interpretation guide* |
| - Experience of ICS side-effects:   - Patient can specifically name one or more side-effects (-):     - Side-effects mentioned in ICS medication information leaflets and summary of product characteristics.     - Other side-effects believed to be caused by ICS according to the patient. | | Mentioning one or more: -  Unclear/comparable answer: +  No side-effects: ++  Min. score: -; Max. score: ++ |
| - The side-effects experienced by ICS reduce the daily functioning of the patient:   - Patient can specifically name one or more of the following (-):     - Has an effect on job/work performance; occasionally stops using the inhaler because of the occurrence of side-effects; consults healthcare professionals for the occurrence of side-effects.     - Other effects of ICS mentioned, that impact the patient’s daily functioning negatively. | | Mentioning one or more: -  Unclear/comparable answer: +  No reduction daily functioning: ++  Min score: -; Max. score: ++ |
| *Final interpretation* | | *Total score* |
|  | Barrier not present: 3-4 plusses. | 0-4 plusses |
|  | Barrier present: 0-2 plusses. |  |
| **TDF-domain: Emotion** | | |
| **Barrier: Concerns about ICS inhaler** | | |
| *Coding components* | | *Interpretation guide* |
| - Concerns about the (regular) occurrence of unwanted side-effects of ICS use:   - Patient can specifically express concerns on one or more of the following (-):     - Side-effects mentioned in ICS medication information leaflets and summary of product characteristics.     - Other side-effects believed to be caused by ICS according to the patient. | | Mentioning one or more: -  Unclear/comparable answer: + No concerns: ++  Min score: -; Max. score: ++ |
| - Concerns about long-term unwanted side-effects:   - Patient can specifically name one or more of the following (-):     - Uncertainties about long-term effects; dependence on ICS; ICS less effective with regular use/development of resistance/stops once in a while to combat resistance.     - Other long-term side effects believed to be caused by ICS according to the patient. | | Mentioning one or more: -  Unclear/comparable answer: +  No concerns: ++  Min score: -; Max. score: ++ |
| - Concerns about the regular use of ICS inhalation/adherence to daily treatment regimen:   - Patient can specifically name one or more of the following (-):     - Time consuming/takes a while to inhale; reduction of freedom; not pleasant to take so much medication; concerns that side-effects occur with regular intake. | | Mentioning one or more: -  Unclear/comparable answer: +  No concerns: ++  Min score: -; Max. score: ++ |
| *Final interpretation* | | *Total score* |
|  | Barrier not present: 5-6 plusses. | 0-6 plusses |
|  | Barrier present: 0-4 plusses. |  |
| **Barrier: Social discomfort of inhaling with ICS in public** | | |
| *Coding components* | | *Interpretation guide* |
| - The experience of social discomfort when inhaling ICS in public:   - Patient can specifically name one or more of the following (-):     - Stands to the side when inhaling/separates from the group/goes to a toilet; patient does not want to burden others; does not want to receive questions or startle bystanders and/or does not want to show he/she has a condition.     - Other reasons depending on the experience of the patient. | | Mentioning one or more: -  Unclear/comparable answer: +  No social discomfort: ++  Min score: -; Max. score: ++ |
| - Social discomfort prevents the patient from inhaling ICS in public:   - Patient can specifically name one or more of the following (-):     - Does not inhale in public when necessary, withdraws from the group or public for inhalation. | | Mentioning one or more: -  Unclear/comparable answer: +  Does not avoid public inhalation: ++  Min score: -; Max. score: ++ |
| *Final interpretation* | | *Total score* |
|  | Barrier not present: 3-4 plusses. | 0-4 plusses |
|  | Barrier present: 0-2 plusses. |  |
| **TDF-domain: Skills** | | |
| **Barrier: Understanding and application of ICS inhaler techniques** | | |
| *Coding components* | | *Interpretation guide* |
| - Understanding of the instructions on ICS inhalation:   - Patient can specifically name one or more of the following points about receiving *good/adequate* information/instructions on ICS inhalation (++):     - Verbal explanation and/or written information received from a healthcare provider; patient searched for additional information/inhalation video’s on the internet; obtained (repeated) practical instruction(s) from a healthcare provider; indicates the usefulness of inhalation with the step-by-step plan of an inhalation instruction; indicates personal improvements in inhalation technique obtained through instructions; receives information from asthma foundations. | | Mentioning one or more: ++  Unclear/comparable answer: +  Did not receive good/adequate information: -  Min score: -; Max. score: ++ |
| - Proper use of the ICS inhaler with the instructions provided:   - Patient can specifically name one or more of the following (++):     - Instructions/explanations have led to good results/good use; patient provides an overview of the steps applied with inhaling; mentions no difficulties. | | Mentioning one or more: ++  Unclear/comparable answer: +  No proper use: -  Min score: -; Max. score: ++ |
| - The patient experiences difficulties in inhaling properly with ICS independent on receiving good/adequate information/instructions:   - Patient can specifically name one or more of the following (-):     - No dose count available on inhaler device (+; dependent on interpretation of the patient given); inhaler disk does not stop spinning and therefore may be empty (+; dependent on interpretation of the patient given); periods when asthma symptoms are more present and therefore more difficult to inhale (+; dependent on interpretation of the patient given; logic consequence of asthma attacks); other difficulties mentioned by the patient concerning inhalation. | | Mentioning one or more: -  Unclear/comparable answer: +  No difficulties: ++  Min score: -; Max. score: ++ |
| *Final interpretation* | | *Total score* |
|  | Barrier not present: 5-6 plusses. | 0-6 plusses |
|  | Barrier present: 0-4 plusses. |  |
| **TDF-domain: Memory, attention and decision process** | | |
| **Barrier: (Un)Conscious adherence to prescribed ICS medication regimen.** | | |
| *Coding components* | | *Interpretation guide* |
| - Conscious or unconscious (non)adherence to ICS medication regimen:   - Patient uses ICS inhaler every day (++); not every day (-)   - Seasonal asthma: every day (++); not every day (-)   - As needed with occurring symptoms: not every day (-) | | Use of ICS every day: ++  Unclear/comparable answer: +  Use of ICS not every day: -  Min score: -; Max. score: ++ |
| - Conscious compliance to ICS medication regimen:   - Patient does not use the ICS inhaler as agreed on with the practitioner by naming one or more of the following causes (-):     - Beliefs of the patient e.g. the patient doesn’t belief he/she needs the ICS inhaler as prescribed; the patient does not inhale when symptoms are not present; worries/concerns e.g. the patient does not like to take so much medication on a daily basis; side-effects prevent the daily use of ICS; misconception/insufficient knowledge e.g. the patient does not understand the benefits of ICS inhalation according to the prescribed regimen; the prescribed treatment regimen affects the daily functioning. | | Use of ICS not as agreed: -  Unclear/comparable answer: +  As agreed: ++  Min score: -; Max. score: ++ |
| - Unconscious compliance to ICS medication regimen:   - Patient often forgets to use ICS medication by naming one or more of the following causes (-):     - Often refers to a lack of structure/routine: irregular working hours/work shift/study hours or work, rushing/busy, frequent travelling, frequent partying or going out; increased responsibilities: including family/children or being cautious of intake because of children; excessively tired or mental problems; consuming too much alcohol; patient attaches little importance or value to use ICS. | | Often forgets use of ICS: -  Unclear/comparable answer: +  Does not forget use of ICS: ++  Min score: -; Max. score: ++ |
| *Final interpretation* | | *Total score* |
|  | Barrier not present: 5-6 plusses. | 0-6 plusses |
|  | Barrier present: 0-4 plusses. |  |
| **TDF-domain: Memory, attention and decision process** | | |
| **Barrier: Shared treatment decision making** | | |
| *Coding components* | | *Interpretation guide* |
| - Healthcare providers have exchanged relevant information about the optimal way of treating asthma with ICS according to the patient:   - A clear (++), limited (+) or no (-) explanation was received about asthma and its treatment. | | Information provided: ++  Limited or unclear/comparable answer: +  No information provided: -  Min score: -; Max. score: ++ |
| - Preferences, complaints and wishes of the patient concerning the ICS inhaler have been discussed:   - Patient can specifically provide information on one or more of the following:     - Resistance against the asthma chamber; uniformity in dosage form of asthma medication; force of inhalation; ease of use. | | Preferences/complaints/wishes have been taken into consideration: ++  Limited or unclear/comparable answer: +  Not taken into consideration: -  Min score: -; Max. score: ++ |
| - The patient feels involved with the current ICS treatment strategy:   - Patient acknowledges the involvement on one or more of the following and is positive about it (++):     - Preferences are included in treatment strategy; complaints are heard and taken into consideration; shared decision on chosen treatment strategy. | | Mentions the involvement: ++  Limited or unclear/comparable answer: +  Not involved: -  Min score: -; Max. score: ++ |
| *Final interpretation* | | *Total score* |
|  | Barrier not present: 5-6 plusses. | 0-6 plusses |
|  | Barrier present: 0-4 plusses. |  |
| **TDF-domain: Behavioral regulation** | | |
| **Barrier: Existence of structure in ICS medication intake.** | | |
| *Coding components* | | *Interpretation guide* |
| - Inhalation with ICS takes place at a fixed time of the day:   - Patient can specifically name one or more of the following (++):     - In the morning and evening; fits into the patient’s daily routine/consistent use/routine/in the system; when brushing teeth; ICS is placed in a fixed spot; fixed time; are in plain sight; keeps a diary/table, mobile phone alarm notification; someone in the family reminds the patient; symptoms act as a reminder; patient attaches great importance/value to use of ICS.   - Patient has no fixed time of the day for inhaling and specifically names one or more of the following reasons (-)     - Irregular working hours/work shift/study hours or work obligations; rushing or very busy; frequent travelling; frequent partying or going out; no fixed place; no fixed time; not in plain sight; increased responsibilities: family, children (caution with intake); patient attaches little importance or value to use ICS. | | Mentioning one or more points concerning fixed time of intake: -  Unclear/comparable answer: +  Mentioning one or more reasons concerning no fixed time of intake: ++  Min score: -; Max. score: ++ |
| *Final interpretation* | | *Total score* |
|  | Barrier not present: 2 plusses. | 0-2 plusses |
|  | Barrier present: 0-1 plusses. |  |
