# Supplementary material for: Respiratory Adherence Care Enhancer Questionnaire: Identifying Self-Management Barriers of Inhalation Corticosteroids in Asthma
Source: Front Pharmacol. 2021 Dec 22;12:767092. doi: 10.3389/fphar.2021.767092 (PMC8729223; doi:10.3389/fphar.2021.767092)
Supplement: Supplementary file 3 [file Image1.PDF]

Supplementary Material

1 Supplementary figure

1.1 Overview of the mixed-methodology steps

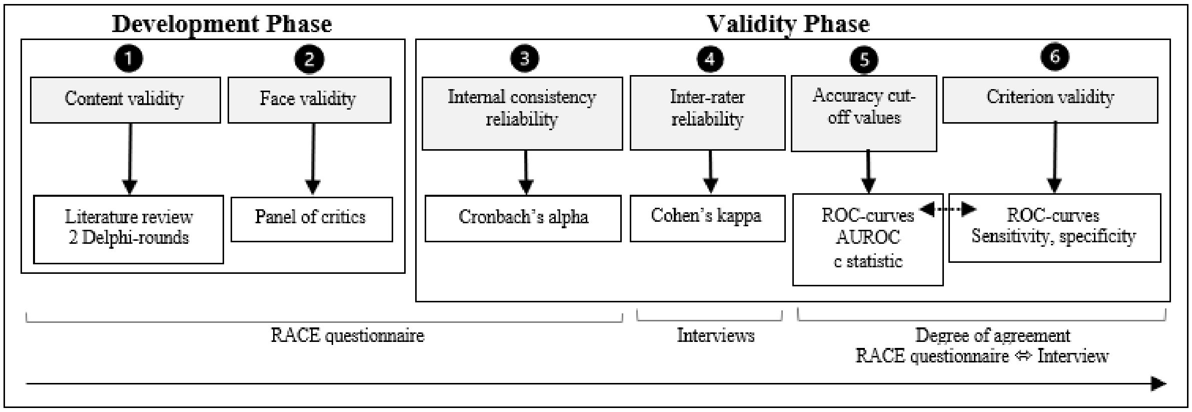

Overview of the mixed-methodology steps applied for the development and validation of the RACE questionnaire with its associated analysis tests. The numbers 1-6 indicate the steps taken in the development and validation of the RACE questionnaire. Steps 1 and 2 comprise the development phase of the RACE questionnaire which consists of the content validity and face validity. Steps 3 to 6 demonstrate the validity phase of the RACE questionnaire which consists of the internal consistency reliability test, the inter-rater reliability test of the interview data, the accuracy of the cut-off values and the criterion validity as final step.

Abbreviations: RACE: Respiratory Adherence Care Enhancer; ROC: Receiver Operating Characteristic; AUROC: Area Under the ROC.
